# Laparoscopic insertion of pelvic tissue expander to prevent radiation enteritis prior to radiotherapy for prostate cancer

**DOI:** 10.1186/1748-717X-6-47

**Published:** 2011-05-14

**Authors:** Gary D McKay, Karen Wong, Daniel R Kozman

**Affiliations:** 1 Department of Colorectal Surgery, Bankstown Hospital, Eldridge Rd, Bankstown, NSW, Australia; 2 Department of Radiation Oncology, Liverpool Hospital, Goulburn St, Liverpool, NSW, Australia

## Abstract

Radiation enteritis is a significant complication of external beam radiotherapy (EBRT) to the pelvis, particularly in patients having high dose radiotherapy (>80 Gy) and in those with a low pelvic peritoneal reflection allowing loops of small bowel to enter the radiation field. Laparoscopic insertion and subsequent removal of a pelvic tissue expander before and after external beam radiotherapy is a relatively convenient, safe and effective method for displacing loops of bowel out of the pelvis. We report on a patient with prostate cancer who ordinarily would not have been a candidate for EBRT due to loops of bowel low in the pelvis. With laparoscopic insertion and subsequent removal of a tissue expander, he was able to have radiotherapy to the prostate without developing radiation enteritis.

## Introduction

Prostate cancer is the second most common cancer in men. With the increasing use of primary radiotherapy for prostate cancer and improved survival, chronic radiation enteritis is an increasing problem occurring in over 20% of patients[1]. The very high doses of 80 Gy radiotherapy required for prostate cancer, which is double that given for most other pelvic malignancies, puts those patients with a low peritoneal reflection and low-lying loops of small bowel in the pelvis, at particular risk of radiation enteritis.

Laparoscopic insertion and subsequent removal of a tissue expander before and after radiotherapy is a relatively convenient and minimally invasive procedure that may be an option for displacing loops of bowel from the radiation field.

## Case Presentation

**Figure 1:**
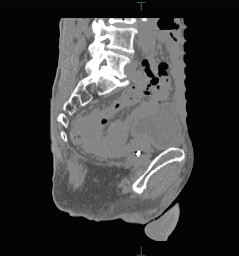
**Planning sagittal CT in 75 year old man with prostate cancer**. Opaque planning fiducial marker visible in prostate with adjacent loops of small bowel within the planned radiation field.

The patient was a 75 year old man with prostate cancer, confirmed by FNA to investigate a raised PSA. He had stage 2 disease with a Gleason score of 3+4, and required primary radiotherapy. He was relatively fit and healthy, with a BMI of 29. He had a previous upper midline laparotomy for gastric lymphoma, and open appendicectomy for perforated appendicitis, and a laparoscopic cholecystectomy. Radiation planning CT demonstrated a low pelvic peritoneal reflection with loops of bowel in the pelvis within the planned radiation field of the prostate (Figure 1).

**Figure 2:**
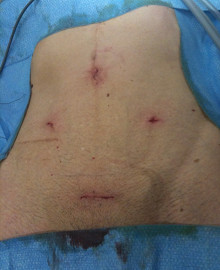
**Four incisions used for laparoscopic insertion of a tissue expander in the pelvis: 12 mm umbilical, 20 mm pfannenstiel, and 5 mm iliac fossa incisions**.

These loops of bowel would not move out of the planned radiation field despite several manoeuvrers including extreme prone and Trendelenburg positioning, bladder filling, and use of an open table-top device (belly board). Laparoscopic insertion of a tissue expander into the pelvis to displace loops of bowel was his only option. No bowel prep was required. A 12 mm infra-umbilical incision was made and an open Hasson technique used to achieve pneumoperitoneum, with the placement of a 12 mm port at the umbilicus and 5 mm ports in both iliac fossa (Figure 2). Adherent loops of bowel from previous surgery were divided by scissored dissection.

**Figure 3:**
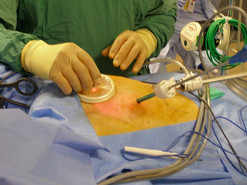
**Insertion of tissue-expander through medium Alexis^® ^ring retractor in the 20 mm supra-pubic incision: this was facilitated by prior lubrication with water-soluble K-Y Jelly^®^**.

In lithotomy and steep Trendelenburg positioning, the suprapubic incision was dilated up to 20 mm with the aid of a medium Alexis^® ^wound retractor (Figure 3).

**Figure 4:**
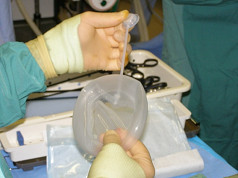
**A 500 ml TRD Nagor^® ^silicone tissue expander with attached inflation port: this allows for filling with normal saline**.

A TRD 500 ml Nagor^® ^tissue expander made of silicone with attached silicone tubing (Figure 4) was then rolled tight, lubricated with water soluble hydroxyethylcellulose and glycerine based (K-Y Jelly^®^) lubricant and inserted via the 20 mm suprapubic port and placed laparoscopically in the pelvis, leaving the normal-saline inflation port attached externally.

**Figure 5:**
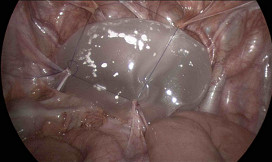
**Tissue expander secured in pelvis with dissolvable running 3/0 polydiaxanone (PDA) purse string stitched to peritoneum of sacral promontory, anterior and pelvic side walls: the expander was subsequently filled with normal saline until it began to bulge against the retaining stitch**.

A running dissolvable 3/0 polydiaxanone (PDA) purse-string stitch was then sutured to the peritoneum of the sacral promontory, and anterior and side walls of the pelvis below the level of the common iliacs, and tied snug to keep the expander in the pelvis. With a Huber^® ^needle inserted into the inflation port, the tissue expander was then filled with 350 ml of normal saline until the expander began to bulge against the retaining stitch (Figure 5).

**Figure 6:**
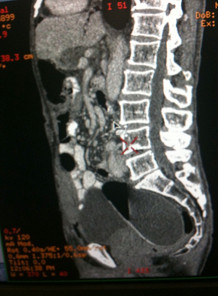
**CT following tissue expander insertion**. The loops of bowel are now well out of the pelvis and planned radiation field.

The abdomen was then deflated of gas and the fascia of both the Pfannenstiel and umbilical port closed. The port of the expander was then placed in a small subcutaneous pocket and sutured to the fascia of the anterior abdomen. Skin incisions were closed in the usual manner. Subsequent CT confirmed adequate placement of the expander device in the pelvis with loops of bowel now well out of the pelvis and the planned radiation field (Figure 6).

His recovery was uneventful being discharged home without complication after opening his bowels. Two weeks later he went on to have external beam radiotherapy to his prostate (80 Gy in 39 fractions over 8 weeks), achieving a good response without any side effects or symptoms. Repeat CT prior to removal of the expander showed a well placed expander within the pelvis, with no evidence of radiation injury to small bowel or the prosthesis.

The tissue expander was removed laparoscopically 6 weeks after completing radiotherapy using the same initial incisions, with a good cosmetic result. There were no adhesions to the silicone implant, and the PDA retaining string was intact, but easily broke with a gentle tug. These two factors facilitated its easy laparoscopic removal.

## Discussion

Radiation enteritis causes considerable disability, and in many cases can be avoided. Any patient having external beam pelvic radiotherapy should have a planning CT, with particular attention given to those patients with a low peritoneal reflection, and loops of bowel within the planned radiation field. Methods of reducing injury to small bowel include multi-field conformal therapy with prior three dimensional planning where the profile of the radiation beam is shaped to fit the target. The delivery of intensity-modulated radiotherapy can also be adjusted and improves the ability of treatment volumes to conform to the shape of the tumour. Despite these techniques, loops of bowel still occasionally get injured from being in the radiation field. If available, brachytherapy or cryotherapy may be reasonable alternatives to external beam radiotherapy. Where external beam radiotherapy is the preferred or only option, various methods for removing small bowel from the radiation field exist.

Conventional non-operative manoeuvres to remove small bowel from the pelvis at the time of giving radiotherapy include extreme prone or Trendelenburg positioning, bladder distension, abdominal wall compression or the use of an open table-top device (belly board). The response of such manoeuvres is not always reproducible. More extreme measures described only in case reports include the surgical insertion of a peritoneal dialysis catheter and creation of a temporary artificial pneumoperitoneum[2] or ascites with the installation of gas or normal saline into the abdominal cavity[3]. These are time consuming, painful, and need to be repeated, and do not reliable remove bowel from the radiation field.

Early surgical procedures to keep small bowel out of the pelvis were aimed at abdomino-pelvic partitioning, either with the use of native tissue or prosthetic material. Native tissue partitioning frequently involves the use of the peritoneum, bladder, uterine broad ligaments and omentum. In 1979, Freund described suturing the anterolateral peritoneum to the bladder and to the anterior rectum[4]. In women, the uterus and broad ligaments may be used in addition to the posterior tissue. In 1985, DeLuca and Ragins described the omental envelope technique (also called an abomino-pelvic omentopexy)[5], where omentum is draped over the small bowel as an apron, and the lower edge sutured to the sacral promontory. The lateral borders are sutured to the ascending and descending colon. In 1995, Choi and Lee described the omental pedicle hammock technique[6] where a pedicle of omentum based on the left gastroepiploics is sutured circumferentially to the peritoneum at the level of the sacral promontory and umbilicus. This creates a sling, or hammock, which keeps small bowel out of the pelvis. Partitioning with prosthetic material has also been described, and includes the use of absorbable mesh slings.

There are many difficulties with partitioning techniques. Firstly, native tissue is frequently not sufficiently adequate or strong enough to achieve partitioning, and prosthetic materials run the risk of infection or adherence to loops of bowel or the creation of a fistula. Partitioning of the pelvis from the abdomen may also create an empty pelvic space. Loops of bowel may get caught beneath the partition resulting in an internal hernia and obstruction. The cavity beneath the partition may also fill with fluid, and this has the potential to become infected resulting in a chronic pelvis abscess.

Pelvic-space occupying techniques avoid some of the problems inherent to partitioning techniques. In 1984, Russ described using an omental pedicle flap based on the left gastroepiploic vessels, which is placed along the left para-colic gutter with the distal tip packed into the pelvis[7]. This is particularly suitable during open colorectal surgery where mobilisation of the omentum off the colon is required. But this procedure is difficult to perform laparoscopically, and in the thin patient, the omentum is usually frequently not sufficient to fill or reach the pelvis.

Normal saline filled silicone tissue expanders are easy to insert and remove and have the benefit of being non-adherent to both peritoneum and small bowel, as well as radioresistant to degradation, and when filled with normal saline, are similar in density to human tissues, therefore do not alter the isodose distribution of radiotherapy. In 1983, Sugarbaker first described the open insertion of a normal-saline-filled silicone breast implant into the pelvis to exclude small bowel from the pelvis to prevent injury from post-operative radiotherapy[8]. Sugarbaker described covering the implant with a mesh which was sutured to the peritoneum of the pelvic brim to prevent migration or extrusion of the expander. However, over the years, mesh was found to be associated with an increased risk of small bowel adhesions and fistula formation. Therefore, there has been a trend away from the use of mesh, with fixation of the expander to the peritoneum with a suture the commonest and safest method. Some expanders have suture tabs for this purpose. Since Sugarbaker's first description, there have been over 160 reported cases of tissue expanders used in the pelvis or the abdomen to prevent radiation injury to small bowel (Table 1). All of these were inserted at open laparotomy. Lasser first described its use prior to radiotherapy for rectal cancer [9]. Many reported cases involved the use of large tissue expanders for patients requiring post-operative adjuvant radiotherapy for large retroperitoneal sarcomas or gynaecological malignancies. For all of these types of malignancies the radiation dose received was usually less than 50 Gy (Table 1).

**Table 1:** Cases to date of intra-abdominal or pelvic insertion of a normal saline-filled silicone implant prior to external beam radiotherapy

Author	Year	Origin	Number	Indication	Radiotherapy	Site	Insertion & removal	Expander size	fixation	Radiation dose	complications due to prosthesis
Sugarbaker[8]	1983	Washington DC, USA	1	unspecified	adjuvant	pelvis	open	up to 1000 ml	prosthetic mesh to pelvic brim	65 Gy	no

Lasser et al[9]	1986	Paris, France	9	rectal cancer	adjuvant	pelvis	open		?	?	no


Armstrong et al[14]	1990	New York, USA	2	fibrosarcoma	adjuvant	renal bed	open	400-500 ml	none	30 Gy	no

Cuttat et al[15]	1991	Lausanne, Switzerland	4	rectal cancer	adjuvant	pelvis	open	500 ml	?	?	no

Delaloye et al[12]	1994	Vandois, Switzerland	18	cervical cancer	adjuvant	pelvis	open	350-400 ml	absorbable suture	56-60.8 Gy	hydronephrosis (n = 1) constipation 9 (n = 1)

Hoffman et al[18]	1998	Philadelphia, USA	58	sarcomas & endometrial, vaginal, rectal, colon & anal cancer	adjuvant (n = 57) primary (n = 1)	pelvis & lower abdomen	open	550-1,500 ml	absorbable suture	40-50 Gy	abscess (n = 4) fistula (n = 4), extrusion (n = 1)

Sezeur et al[10]	1999	Paris, France	22	retroperitoneal sarcoma and pelvic cancer.	adjuvant	pelvis & abdomen	open	?	?	30-65 Gy	heaviness (n = 1) flank pain (n = 1)

Burnett et al[11]	2000	Los Angeles, USA	7	cervical cancer	adjuvant	pelvis	open	750-1,500 ml	absorbable suture	50.4 Gy	adhesions of bowel to implant (n = 1) pulmonary embolism (n = 1)

Abhyankar et al[13]	2005	Wales, UK	1	rhabdomyosarcoma	adjuvant	right upper abdomen	open	250 ml	mesh	45 Gy	no

Hølmebakk et al[16]	2006	Oslo, Norway	1	Retroperitoneal recurrence of colorectal cancer	adjuvant	pelvic	open	500 ml	sutured & omental sling	50 Gy	no

White et al[20]	2007	Calgary, Canada	33	sarcomas & endometrial, vaginal, rectal & colon cancer	neo-adjuvant (n = 25) adjuvant (n = 1) primary (n = 11)	pelvis & abdomen	open	700 ml	Dexon^® ^mesh	45-50 Gy	Cystitis (n = 1) Ileus (n = 1)

Hong et al[19]	2008	Sydney, Australia	2	retroperitoneal sarcoma & abdominal wall sarcoma	adjuvant	lower abdomen	open	1000 ml	?	50 Gy	no

Angster et al[17]	2010	Baltimore, USA	2	cervical cancer & retroperitoneal sarcoma	adjuvant	pelvis & abdomen	open	400 ml & 500 ml	none	?	no

McKay et al	2011	Sydney, Australia	1	prostate cancer	primary	pelvis	laparoscopic	500 ml	absorbable suture	80 Gy	No

Early experience with tissue expanders found that complications were more common when large expanders where left in the pelvis long term, with the potential for bladder, ureteric and iliac vessel compression. Heaviness is a common complaint of very large expanders[10]. Deep vein thrombosis with pulmonary embolism and constipation due to obstructive defecation have been reported[11,12]. More recent reports using smaller implants show them to be associated with fewer complications[13-17]. Infection, with abscess formation and fistulisation, have been reported to occur in up to 7% of cases[18]. Wound infections associated with large laparotomy incisions are not uncommon, particularly when the incision extends into the radiation field[19]. The other disadvantage of tissue expanders, is that they do very little to prevent radiation injury to the bladder or rectum, with radiation cystitis[20] and proctitis still common complications.

Ours is the first report in the literature of a totally laparoscopic insertion and removal of a tissue expander prior to and following primary prostatic radiotherapy. In this case, a much higher dose of 80 Gy radiotherapy was given. Previous major surgery was not a contraindication to this procedure. In our case, a conventional 500 ml normal-saline-filled silicone tissue expander without suture tabs, was used, and it was kept in the pelvis by means of a polydiaxanone (PDA) purse string suture. This monofilament has a tensile-strength half life of 5 weeks, with significant degradation of the suture at 10-12 weeks. Therefore removal of the expander was performed easily by gentle traction alone. However there is a risk with dissolvable sutures of tissue expander migration, therefore a non-dissolvable suture may also be appropriate. The choice of a conventional sized expander and the avoidance of overfilling were because of literature reports of the risks of ureteric and iliac vessel compression.

The ease, simplicity, reversibility, and minimally invasive nature of laparoscopic tissue expander insertion are its main appeal. It should be considered as an option for excluding small bowel from the pelvis prior to radiotherapy of the prostate.

## Consent

Written informed consent was obtained from the patient for publication of this case report and accompanying images. A copy of the written consent is available for review by the Editor-in-Chief of this journal.

## Competing interests

The authors declare that they have no competing interests.

## Authors' contributions

GM wrote the manuscript, KW did literature review and organised planning CT and radiotherapy; DK inserted and removed the tissue expander and supervised writing of manuscript. All authors read and approved the final manuscript.
